# “Everything has changed”: detention officer roles and recreation time changes due to COVID-19 policies at a Southwest County Jail

**DOI:** 10.1186/s40352-022-00181-x

**Published:** 2022-06-04

**Authors:** Travis Pinn, Heather Williamson, Bethany Robinson, Sara Shuman, Maria Evans, George Pro, Ricky Camplain

**Affiliations:** 1 Center for Health Equity Research, Northern Arizona University, Flagstaff, AZ USA; 2 Department of Health Sciences, Northern Arizona University, 1395 S. Knoles Drive, ARD Building, Suite 140, PO Box 4065, Flagstaff, AZ 86011-4065 USA; 3 Department of Occupational Therapy, Northern Arizona University, Flagstaff, AZ USA; 4 Department of Biological Sciences, Northern Arizona University, Flagstaff, AZ USA; 5 Department of Psychological Sciences, Northern Arizona University, Flagstaff, AZ USA; 6 Department of Health Behavior and Health Education, University of Arkansas for Medical Sciences, Little Rock, AR USA

**Keywords:** Detention officer, Correctional officer, COVID-19, Job duties, Recreation time

## Abstract

**Background:**

The COVID-19 pandemic responses in jails have forced detention officers to adjust how they approach the confinement and care of individuals while they are incarcerated. One aspect of incarceration affected was detention officers’ roles. The aims of this research project were to determine how the COVID-19 pandemic has affected the general duties of detention officers at a Southwest County Jail. Detention officers were recruited via email to participate in an online questionnaire from October to December 2020. Participants answered Likert scale and open-ended questions regarding how the COVID-19 pandemic has affected their job duties. Descriptive statistics and thematic analysis were used to identify themes and patterns in the responses.

**Results:**

Among 24 detention officers, 87% indicated agreement that the COVID-19 pandemic has significantly changed the duties of detention officers at CCDF. The most discussed change was the introduction of a 14-day quarantine process for newly incarcerated individuals. The 14-day quarantine increased the workload of detention officers.

**Conclusion:**

The COVID-19 pandemic responses in jail may have unintended negative consequences for the job duties of detention officers. Current and future pandemic response strategies in jails would benefit from taking staff perspectives into consideration as they are directly impacted by the COVID-19 response strategies put into place. Policy implications are discussed.

## Background

Prisons and jails are hotspots for Coronavirus 2019 (COVID-19) as detention officers are at a high risk of spreading and contracting the disease, due to confined conditions (Saloner et al., 2020). Jail and prison staff contracted COVID-19 at a higher rate than the general population (Jiménez et al., 2020; Nowotny et al., 2021). The high rate of COVID-19 among correctional staff may be due to the unique characteristics of jails that increase the risks associated with the transmission of SARS-CoV-2, the virus that causes COVID-19. Jails are congregate living settings characterized by limited opportunities for physical distancing and lack of resources including COVID-19 testing and personal protective equipment (PPE) (Bick, 2007). In addition, jails involve frequent intakes and releases, which may increase the likelihood of introducing new COVID-19 infections into jails and nearby communities (McMillen, 2020). Substandard health care in jails may also contribute to the spread of the disease among detention officers, incarcerated individuals, and the community (Union, 2020).

Detention officers job duties in jails primarily revolve around overseeing the custody, safety, and security of people incarcerated. Detention officers job duties are shaped in significant ways by the organization’s policies and social climate, including through their stress levels (Morse et al., 2011) and how they approach their job duties (Griffin, 1999, 2001). For instance, Griffin found that a county jail’s social climate influenced use of force in the facility regardless of individual characteristics of detention officers (Griffin, 1999). In general, the occupation of officers working in correctional settings may contribute to elevated stress levels (Kielyl & Hodgson, 1990). Another study found that officers who are exposed to elevated levels of infectious disease during their job duties in prisons were more stressed and more likely to report lower job satisfaction (Hartley et al., 2013).

The stress of detention officers is also affected by increase in stress levels among people incarcerated in the facilities where they work. The COVID-19 pandemic and associated mitigation strategies may contribute to increased mental helath issues among people incarcerated (Barrenger & Bond, 2021). One way to mitigate the mental stresses of being incarcerated may be recreation time, a time dedicated to physical activity and exercise, outside. Thus, recreation time may be an important tool for increasing perceptions of positive relationships between people incarcerated and staff in jails and prisons (Vuk & Doležal, 2020). However, there is limited literature on how new pandemic mitigation policies in jails may impact recreation time, particularly from the perspective of detention officers.

In response to the increased risks posed to correctional staff, various public health strategies to limit the spread of SARS-CoV-2 have been recommended (Centers for Disease Control and Prevention, 2020). Recommendations include the development of protocols for screening new detainees, the use of PPE among detention officers and incarcerated individuals, physical distancing, cleaning and disinfecting areas and surfaces right after use, and restriction of transfers and access for non-essential staff and visitors (Kinner et al., 2020). Other public health experts have recommended depopulating jails, using strict quarantine procedures, and regularly testing both detention officers and incarcerated people (Franco-Paredes et al., 2020).

To our knowledge, no research has examined how preventive responses to the COVID-19 pandemic has affected the working conditions in jails. Thus, our objective was to examine the changes caused by the COVID-19 pandemic on detention officers’ duties in a county jail in Arizona.

## Methods

### Study setting and population

This cross-sectional, web-based study was conducted with detention officers at a Southwest county jail between October 9 and December 1, 2020. The jail is a regional holding facility, that houses a diverse group of people who are sentenced and unsentenced for misdemeanors and felonies (Camplain et al., 2019). Detention officers were recruited to participate in the study via an email from the jail Programs Coordinator. Participation was voluntary and detention officers who completed the questionnaire received a $25 e-gift card for participating. The jail Programs Coordinator followed up his original email with two additional reminder emails for recruitment.

### Questionnaire development

A questionnaire was developed by a team of researchers at the Center for Health Equity Research at Northern Arizona University to examine detention officers’ perceptions of women who are incarcerated and their recreation time behaviors. The questionnaire was developed through an iterative process in which prior systematic observations of recreation time were built upon to assess detention officers’ role in incarcerated women’s physical activity from the detention officers’ point of view (Camplain et al., 2020). For the current study, participants were asked to respond to the following on a 6 point Likert Scale: (1) Has recreation time changed since the COVID-19 crisis began? (2) Have your job duties at the jail changed since the COVID-19 crisis began? Following their responses, participants were additionally asked (1) How has recreation time changed since the COVID-19 crisis began? and (2) How have your job duties at the jail changed since the COVID-19 crisis began? The detention officers were given the option to explain how their job duties have changed in an open-text response in the questionnaire. Finally, the detention officers were asked demographic questions including their gender, race, ethnicity, marital status, age, education level, and length of time as a detention officer.

### Data analysis

Open-ended responses were analyzed using qualitative thematic analysis method.^11^ A study team member first coded responses followed by a review by other members of the team. Coded responses were grouped into larger themes. Demographic characteristics and Likert scale responses were calculated using descriptive statistics, including relative frequencies, medians, and interquartile ranges (IQR).

## Results

Of approximately 50 detention officers at the jail, 24 detention officers participated in the questionnaire. Most participants were male, white, under 35 years of age, non-Hispanic, single, and college-educated (Table 1). The average length of time as a detention officer was 4.8 years.

**Table 1:** Demographic Characteristics of Coconino County Detention Facility Detention Officers (*n* = 24)

	Frequency	Percentage
Gender		
Female	3	12.5%
Male	20	83.3%
Prefer not to answer	1	4.2%
Age		
18–24	9	37.5%
25–34	10	41.7%
35–44	3	12.5%
45–54	2	8.3%
Race		
Other ^a^	4	16.7%
White	20	83.3%
Ethnicity		
Hispanic/Latino	4	16.7%
Non-Hispanic/non-Latino	19	79.2%
Prefer not to answer	1	4.2%
Education Level		
High school diploma or GED	4	16.7%
Some college	10	41.7%
4-year college degree or more	10	41.7%
Marital Status		
Single	13	54.2%
Married	11	45.8%
Length of time as detention officer		
≤ 1 year	5	20.8%
> 1 < 4 years	9	37.5%
> 4 years	9	37.5%
Unknown	1	4.2%
Total	24	100%

^a^Other race includes American Indian/Alaskan Native, Asian or Pacific Islander, Black/African American, and prefer not to answer

### Likert scale responses

**Fig. 1:**
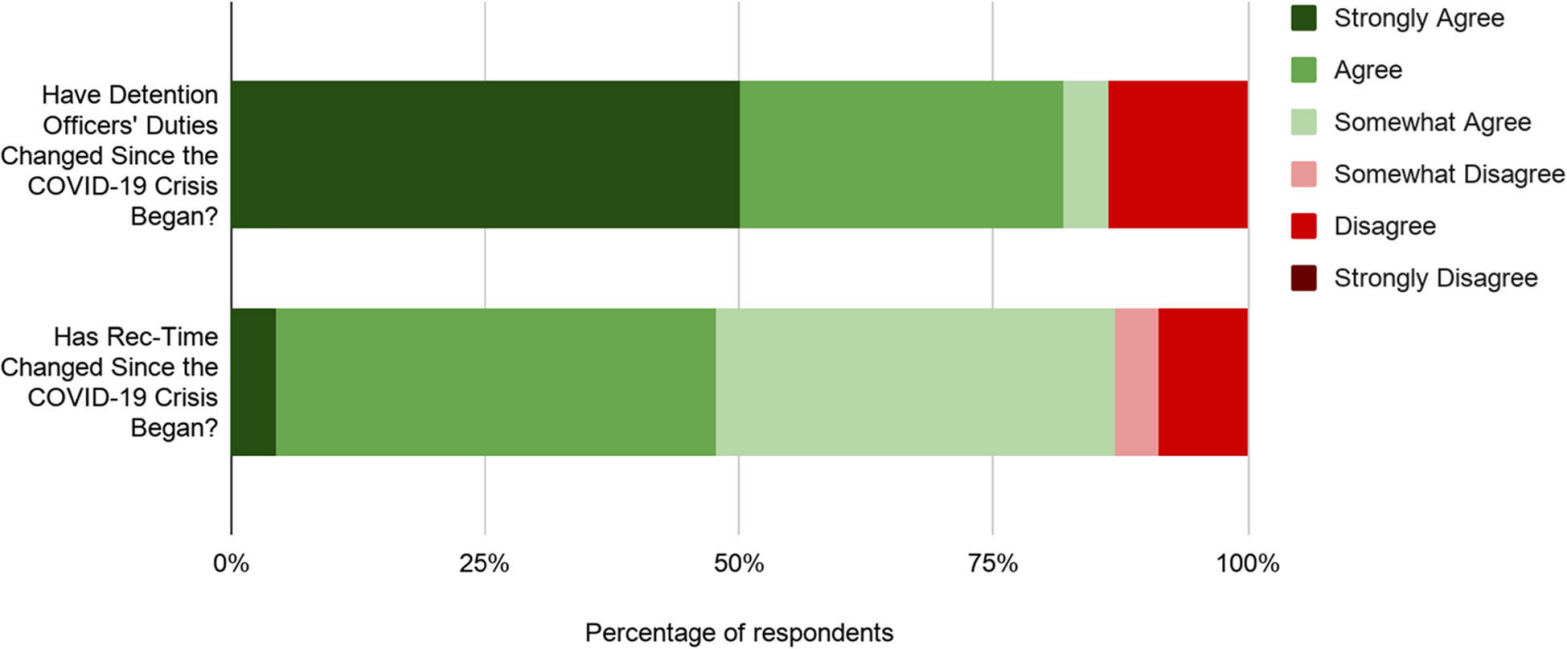
Has COVID-19 Changed Recreation Time or Detention Officers’ Duties?

Among detention officers, there was general agreement that the COVID-19 pandemic had altered both their job duties and recreation time. Almost 90% of detention officers indicated some level of agreement that their job duties changed since the COVID-19 pandemic began with a median score of 6 (IQR = 5,6), indicating most participants strongly agreed (Fig. 1).

Approximately 87% of respondents agreed that recreation time changed since the COVID-19 crises began (Fig. 1). However, the respondents agreed less strongly that recreation time has changed compared to their overall job duties. For instance, only 48% either ‘strongly agree’ or ‘agree’ that their duties around recreation time have changed since the crises began (median = 5, IQR = 4,5) compared to 78% who responded ‘strongly agree’ or ‘agree’ to the question of have their job duties changed since the COVID-19 crisis began.

### Open-ended responses

Among 20 participants, the dominant theme (Theme 1) in responses to “How have your job duties at the jail changed since the COVID-19 crisis began?” was the belief that there were significant changes to their job duties during the COVID-19 pandemic, including changes that have caused them more stress than usual in their work. The most common subtheme (Subtheme 1) in responses to this question was the added job duties related to the 14-day quarantine procedure. Like subtheme 1, the dominant theme (Theme 2) in responses to “How has recreation time changed since the COVID-19 crisis began?” was that quarantine had altered the way that recreation time operated at the jail. Quarantine involves adjusting DO schedule operations to account for recently incarcerated individuals, who are housed in separate housing, are unable to attend recreation time, and are nevertheless required to be supervised. A significant subtheme (Subtheme 2) was new concerns among detention officer about the welfare of quarantined individuals who were not allowed recreation time.

### Theme 1: significant changes to detention officers’ job duties

Changes to job duties among many officers were perceived as significant specifically to their daily tasks with additional strain during working hours therefore to job duty changes. Primarily, detention officers discussed increased job duties in reference to new public health measures, which in turn has caused increase in feelings of stress among officers. The most common public health measure discussed by the officer was “cleaning” or “sanitizing.”

In general, there was agreement that beyond the new cleaning responsibilities, there were a lot of other “extra duties” for detention officers related to “new policies and procedures.” As one officer simply stated, “Everything has changed.” The new non-cleaning duties included taking more and smaller groups of people incarcerated when they need to be transported through the jail, “checking temperatures for all newly booked inmates,” and “officers must spend significantly more time doing medical pass with nurse staff due to the raise in need for inmates to be seen during quarantine.” One officer said that despite the new cleaning, transportation, and medical duties, there has been no “additional support of staffing.” Another explained that in general, “Every process takes a little longer. It is more difficult to keep people separate when necessary.” One officer suggested that additional job duties have made the job “more stressful.” One officer summarized the extent of how changes related to their specific job duties had affected interactions with incarcerated people and his family.

*“Firstly to reduce the spread of COVID, we are required to wear a N-95 mask when interacting with inmates. As the Classification Officer I am responsible for getting every inmate in custody in front of a judge within 24 hours of arrest. (There are some exceptions) When transporting inmates to and from court, before it would be similar to follow the leader with me being the leader along with follow a few of my rules. Now, I need to separate inmates if they are on COVID Isolation (being symptomatic or being exposed to COVID) or regular on top of all the other behavior issues I encounter on a daily basis. With precautions and medical requirements in order to do my job safely for me, staff, other inmates and even my family there has been a lot more required for even the most simplest of tasks.”*

### Subtheme 1: quarantine procedures have changed job duties

Subtheme 1, quarantine, was common among detention officers. For example, additional staff were needed for the quarantine procedure. Part of the quarantine procedure, according to another detention officer, involves supervising single incarcerated individuals during quarantine who “must have an hour out in the dayroom.” The detention officer then explained that after this period, “the officers on the floor will peroxide all the tablets, tables, phones, etc. so the next inmate can come out.” This was echoed by several other officers, who discussed the duty to “sanitize” or “clean” after “quarantines” have used the day room.

Another officer emphasized the significance of the changes, especially how procedures related to quarantine have increased their job duties:

*“It’s been more stressful due to the amount of work we have to do now on top of our other job duties for instance in our housing units we have to sanitize every quarantine dorm after the inmates have time out.”*

### Theme 2: quarantine has changed recreation time

Most respondents to “How has recreation time changed since the COVID-19 crisis began?” discussed that quarantine procedures had impacted recreation time. Detention officers consistently explained that due to the 14-day quarantine period for newly incarcerated individuals, these individuals “don’t get recreation time.” Quarantine had also altered the operation of recreation time schedules. As one detention officer explained: quarantine “has changed how the rec schedule operates.” Another officer revealed, “Fewer people are able to go at once so it’s more difficult to get everyone out regularly.” There was a commonly shared sentiment that these limitations were beyond the individual officers’ control and due to “the jail” or, more specifically, due to the “14 day quarantine.” The above examples indicate that despite fewer individuals attended recreation time, detention officers were required to manage more schedules, requiring extra efforts to mitigate the risk for contracting and spreading COVID-19 to people inside the facility.

### Subtheme 2: concern for welfare due to lack of access to recreation time

Finally, the responses to how COVID-19 impacted recreation time revealed the officers understanding of the dual effects of recreation time. Specifically, the detention officers discussed their understanding of the health and safety purposes of the 14-day quarantine and other measures. On the other hand, they also discussed the unfortunate side-effects of such procedures. It was pointed out in multiple responses that the 14-day quarantine procedure was “for the health and safety of inmates” and “not a punishment.” Such statements indicated that officers felt some shared responsibility to abiding by the quarantine procedure and overcoming unfavorable attitudes towards it. Some officers discussed empathy for the incarcerated individuals going through quarantine. As one officer put it: incarcerated individuals have access to recreation time after the quarantine period,

*“However when inmates come into the jail and stay after court, the first few days it could be beneficial for them to go to the gym to improve mood, outlook on life or their case and help the forms behavior in general. This has recently stopped due to COVID.”*

Such a statement shows not only a willingness to abide by COVID-19 safety procedures, but also a realization of the mental health and social benefits of physical activity for incarcerated individuals early in their stay at the jail.

## Discussion

The objective of this research was to understand detention officers’ perceptions about how their general job duties, particularly around recreation time, have changed since the COVID-19 pandemic began at a Southwest county jail. There was general agreement among detention officers that the COVID-19 pandemic has changed both the job duties for detention officers as well as recreation time.

Job duties went through dramatic changes because of the jail’s response to the COVID-19 pandemic. The most common way that detention officers viewed how their job duties had changed was through the implementation of the 14-day quarantine procedure for newly incarcerated individuals. Because of this new policy, the detention officers discussed that they worked more closely with medical staff at the jail, regularly cleaned quarantine dorms, took extra precautions (such as wearing PPE) while interacting with quarantined individuals, and thoroughly cleaned each quarantined dorm after the quarantine period was over. There was general agreement that detention officers’ new job duties involve extra tasks while many previously existing tasks now took longer. They also pointed out that this increase in job demands had not been met with increase in staff support.

The concerns described by detention officers in this study confirm the predictions offered in the literature. For instance, public health researchers predicted that the COVID-19 pandemic responses in correctional facilities would lead to staff shortages and increases in job demands and duties (Montoya-Barthelemy et al., 2020). Responses in our study indicated that detention officers felt that the jail was understaffed to handle increased job duties they experienced causing the job to be overwhelming. As one officer put it, “Everything has changed.” With an increase in job duties among detention officers that results in staff being overwhelmed and stressed without a simultaneous increase in resources, there are implications for officer wellbeing. In addition to overburdening detention officers and increasing the chance of burnout and poor mental health outcomes (Ferdik, 2010; Montoya-Barthelemy et al., 2020), detention officers may spend what little time is dedicated to ensuring the health and wellbeing of individuals incarcerated in other tasks. With excess job duties that staff may not be trained for, detention officers may be more distracted and not as attentive to the needs of individuals incarcerated, including, but not limited to, determining if individuals are sick, in a mental health crisis, or if there is a safety incident or issue. Additionally, detention officers may not have time to transport individuals to provide materials for what little programming and opportunities that are still available during the COVID-19 pandemic, such as recreation time. Our study was limited to the detention officer perspective and did not explore health and safety impacts of increases in job duties on individuals incarcerated.

To note, we did not include questions regarding changes in the variety of tasks detention officers were required to perform during a typical day beyond recreation time. The officers view that people incarcerated may be attending less recreation time due to the 14-day quarantine needs to be further explored. Questionnaire responses revealed only a limited understanding of the detention officers’ perspectives on the significance of the reduced recreation time for people incarcerated due to the pandemic response strategies.

Other public health researchers examining the potential effects of COVID-19 responses have suggested that agitation will occur among incarcerated individuals in quarantine (Montoya-Barthelemy et al., 2020). Since physical activity is known to have positive effects on agitation (Alessi et al., 1999; Hartescu et al., 2015), physical activity opportunities for those in the early period of their incarceration could have positive health impacts for incarcerated individuals as well as on the staff-inmate relationship and general jail climate.

## Conclusions

The unintended consequences of important public health measures in jails during the COVID-19 pandemic have not received much attention, particularly the impact of the increase in job duties due to increased disease mitigation efforts. Detention officer perspectives on how necessary public health measures without additional resources in jails are playing out in real-time are important to determine the unintentional outcomes in jail due to the COVID-19 pandemic. We determined that the COVID-19 pandemic has increased job duties among detention officers and has revoked certain privileges from individuals incarcerated during quarantine, such as recreation time. Because there seems to be no additional resources provided to these facilities in a time where both staff and individuals incarcerated are at an increased risk of disease, necessary mitigation procedures to prevent the spread of SARS-CoV-2 may not have been implemented the way they were intended. The policy implications for our study indicate that more staff support through increased resources and staffing may be necessary to meet the increase demands on detention officers. In addition, altering policy to ensure that incarcerated individuals in quarantine have access to physical activity may produce positive results for both incarcerated individuals and detention officers. For instance, when incarcerated individuals participate in physical activity, they may experience regulation of body temperature, adrenal activity, and neurotransmission of noradrenaline and dopamine. These regulations can contribute to short-term tranquillizing effects, stress adaptation, and improved mood. Thus, individuals incarcerated may be less likely to present behavioral problems for detention officers. Future research would do well to consider how unintended negative consequences of responses to COVID-19 in jails could be mitigated through such policy changes.

## Data Availability

The datasets generated during and/or analyzed during the current study are not publicly available due to human subjects protections but are available from the corresponding author on reasonable request.
